# Maternal Methyl Donors Supplementation during Lactation Prevents the Hyperhomocysteinemia Induced by a High-Fat-Sucrose Intake by Dams

**DOI:** 10.3390/ijms141224422

**Published:** 2013-12-16

**Authors:** Paul Cordero, Fermin I. Milagro, Javier Campion, J. Alfredo Martinez

**Affiliations:** 1 Department of Nutrition, Food Science and Physiology, University of Navarra, 31008 Pamplona, Spain; E-Mails: pcordero@alumni.unav.es (P.C.); fmilagro@unav.es (F.I.M.); jcampion@unav.es (J.C.); 2 CIBERobn, Physiopathology of Obesity and Nutrition, Centre of Biomedical Research Network, 29029 Madrid, Spain

**Keywords:** cardiovascular, HFS diet, homocysteine, maternal programming, methyl donors, obesity

## Abstract

Maternal perinatal nutrition may program offspring metabolic features. Epigenetic regulation is one of the candidate mechanisms that may be affected by maternal dietary methyl donors intake as potential controllers of plasma homocysteine levels. Thirty-two Wistar pregnant rats were randomly assigned into four dietary groups during lactation: control, control supplemented with methyl donors, high-fat-sucrose and high-fat-sucrose supplemented with methyl donors. Physiological outcomes in the offspring were measured, including hepatic mRNA expression and global DNA methylation after weaning. The newborns whose mothers were fed the obesogenic diet were heavier longer and with a higher adiposity and intrahepatic fat content. Interestingly, increased levels of plasma homocysteine induced by the maternal high-fat-sucrose dietary intake were prevented in both sexes by maternal methyl donors supplementation. Total hepatic DNA methylation decreased in females due to maternal methyl donors administration, while *Dnmt3a* hepatic mRNA levels decreased accompanying the high-fat-sucrose consumption. Furthermore, a negative association between *Dnmt3a* liver mRNA levels and plasma homocysteine concentrations was found. Maternal high-fat-sucrose diet during lactation could program offspring obesity features, while methyl donors supplementation prevented the onset of high hyperhomocysteinemia. Maternal dietary intake also affected hepatic DNA methylation metabolism, which could be linked with the regulation of the methionine-homocysteine cycle.

## 1. Introduction

Maternal environment during perinatal periods affects metabolic and physiological features of the offspring in adulthood [1]. One of the external factors implicated in newborns programming is maternal nutrition [2,3]. Pioneer epidemiological studies reported higher rates of cardiovascular diseases during adult life of children born around historical famines as Second World War Dutch famine [4] or Chinese Great Famine during 1959–1961 [5]. These investigations promoted the coining of the Developmental Origins of Health and Disease theory (DOHaD), which suggested that there are critical periods during perinatal life, in which maternal nutrition and other environmental factors induce changes in offspring metabolism and influence the susceptibility to suffer chronic diseases during the adult life [6].

Subsequently, in order to assess the molecular mechanisms underlying DOHaD theory, the study of animal models has been settled as an adequate approach for the control of confounding factors, timing and to overcome ethical dilemmas [7,8]. Thus, epigenetic reactions have been established as putative regulatory mechanisms implicated in perinatal programming [1,9,10]. The term epigenetics refers to marks over the DNA without changes in the nucleotide sequence, affecting gene expression and potentially transmitted to the descendants [11]. One of the most studied epigenetic reactions is the methylation of CpG sites (cytosines followed by a guanine) at gene promoter regions, which can affect the binding of transcriptional factors to the DNA in the process of gene translation on phenotype [11]. Some of these reactions are mediated by DNA methyltransferases (Dnmts), whose activity depends on the availability of methyl donor groups and are a part of the methionine-homocysteine cycle [12]. In this context, there are substrates implicated in this cycle, which can be provided by the diet, such as a cocktail including choline, betaine, folic acid and vitamin B_12_, which may prevent the transgenerational amplification of obesity by epigenetically silencing the murine agouti transposon [13]. These investigations demonstrated the potential regulatory role on gene expression of these substrates through DNA methylation. Other studies also demonstrated that reductions in the availability of vitamin B_12_, folic acid and methionine during the periconceptional time period leads to epigenetic modifications in the offspring and, consequently a phenotype of obesity and an associated high blood pressure throughout the life-course [14]. Furthermore, dietary restriction of some of these molecules in adult animals has been used to induce animal models of non-alcoholic fatty liver disease (NAFLD) as well as advanced states of steatohepatitis, cirrhosis or even hepatic carcinoma [15]. Contrariwise, novel studies have described the protective effect of these compounds on hepatic obesogenic diet-induced fat accumulation in adult male and female Wistar rats [16–18]. These studies described changes in liver transcriptomic and epigenetic profiles due to methyl donor supplementation, suggesting that this organ could be one of the main targets for methyl donor substrates. Moreover, the liver is considered a major site of methionine cycle activity, playing an important role in homocysteinemia regulation [19]. Indeed one carbon metabolic pathway can also regulate plasma homocysteine levels, which are closely associated with cardiovascular diseases [20]. Some studies have been focused on the supplementation with specific methionine-homocysteine cycle-related compounds during adult life for the control of plasma homocysteine levels [21].

In this context, as maternal diet could affect offspring health during the whole life, this stage could be an adequate moment for performing interventions to prevent chronic diseases in the next generation. Thus, the aim of this research was to investigate the potential role of methyl donor supplementation during lactation in the early prevention of cardiovascular risk, by reducing hyperhomocysteinemia in the offspring of rats fed an obesogenic diet.

## 2. Results and Discussion

### 2.1. Phenotypical and Body Composition Characteristics

In order to minimize previous confounding factors to the maternal dietary intake during lactation, we investigated the possible differences related with the pregnant status (Table 1). Previous studies have highlighted the influence of these features on offspring growth and development along life course [22–24]. In this way, at baseline none of the four groups of dams presented phenotypical and pregnancy-related differences in any of the analyzed variables, which included body weight before pregnancy, weight gain during pregnancy and after birth as well as intrauterine number of total, male and female pups.

**Figure 1.**
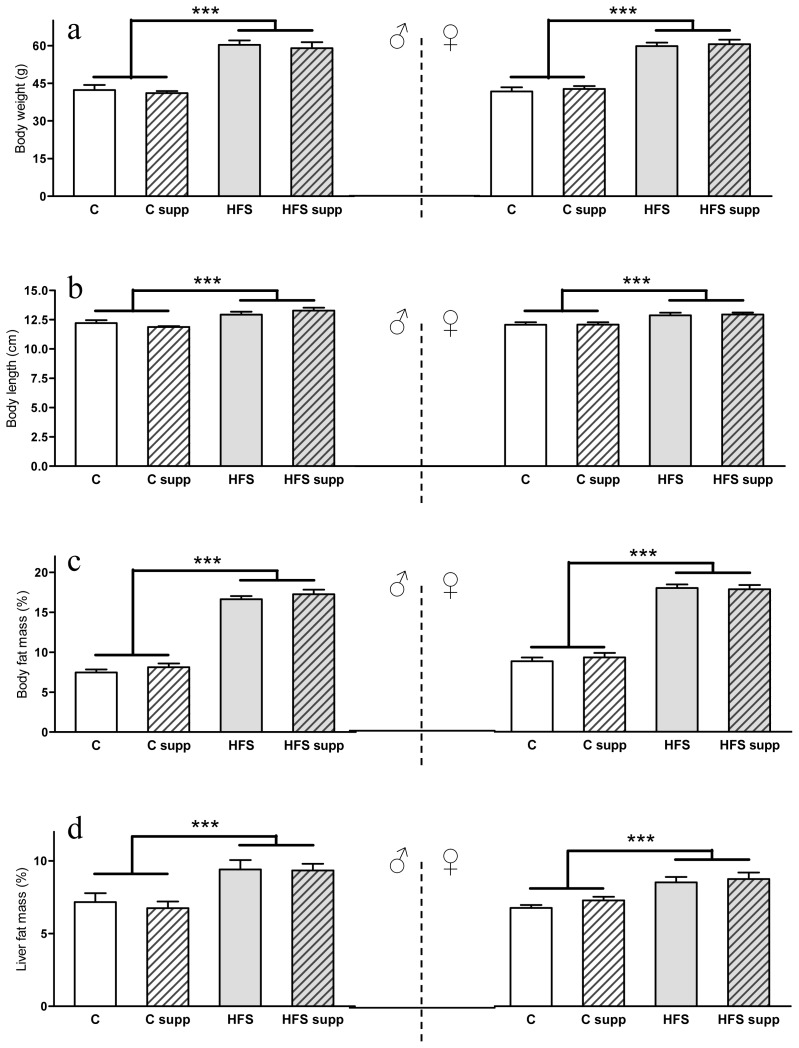
Body weight (**a**); length (**b**); body fat mass (**c**) and liver fat mass (**d**) from male and female offspring depending on maternal nutrition during lactation. Data are reported as means ± SE (*n* between 6 to 8 from each experimental group). C, control; C supp, control supplemented; HFS, high-fat-sucrose; HFS supp, high-fat-sucrose supplemented; ***p < 0.001.

**Figure 2.**
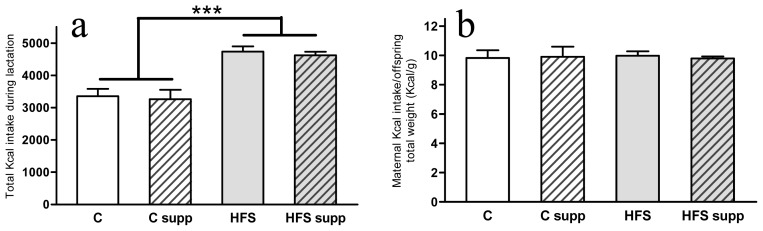
Maternal energy intake during lactation (**a**) and energy consumption per offspring weight (**b**). Data are reported as means ± SE (*n* = 8 from each experimental group). C, control; C supp, control supplemented; HFS, high-fat-sucrose; HFS supp, high-fat-sucrose supplemented; ***p < 0.001.

As expected, the maternal HFS obesogenic dietary feeding induced an obese phenotype in the offspring in both sexes (Figure 1) characterized by increased body weight (43% in both male and females; *p* < 0.001), length (9% in males and 7% in females; *p* < 0.001), body fat mass (118% in males and 98% in females; *p* < 0.001) and liver fat content (35% in males and 24% in females; *p* < 0.001). Although changes in the dietary pattern could affect feeding behavior, offspring from high-fat-sucrose fed dams presented higher weight in both sexes since day 7 of life (*p* < 0.001). On the other side, we have previously reported on adult male and female Wistar rats the protective effect on liver fat accumulation of methyl donor supplementation [17,18]. However, no differences were found as a result of maternal supplementation with methyl donors, which could be the result of the early stage of liver steatosis, the physiological status of the growth period or the different method of administration of these compounds. Factors affecting the basis of offspring metabolic encoding, perinatal adverse nutritional feeding and early postnatal diet, are involved in the onset of many chronic adult diseases, such as obesity, insulin resistance or hypertension [1,16,24]. Thus, maternal nutrition could program gene expression patterns to the offspring that persist into adulthood and may contribute to the appearance of typical metabolic syndrome features such as hypertension, insulin resistance, hyperlipidemia and abdominal obesity [24]. On the other hand, total maternal energy intake (Kcal) during the lactation period (Figure 2a) was higher when mothers were fed the HFS diet (40%; *p* < 0.001). Interestingly, this extra energy intake was mainly spent in offspring growth (Figure 2b), which could be a natural adaptive mechanism for progeny survival expectance [25].

### 2.2. Plasma Biochemical Markers

The alteration of plasma lipid profile due to an obesogenic diet intake is a key feature from experimental models of diet-induced obesity, both on adult [26] or maternal perinatal feeding [24]. Regarding male offspring biochemical plasma variables (Table 2), total and HDL cholesterol levels were augmented (23% and 20% respectively; *p* < 0.001) in the HFS groups. Furthermore, maternal HFS feeding also increased triglycerides (50%; *p* < 0.01) and free fatty acid (60%; *p* < 0.01) plasma values. Female offspring showed similar alterations due to maternal HFS feeding, which included higher total (24%; *p* < 0.001) and HDL cholesterol (13%; *p* < 0.05) levels, and free fatty acids (55%; *p* < 0.01), as well as a trend to increase LDL cholesterol (20%; *p* < 0.1) and triglyceride (58%; *p* < 0.1) plasma levels. However, we have to be cautious as plasma was obtained without an overnight fasting, but animals were fasted about 4 h before the sacrifice.

On the other hand, maternal supplementation with methyl donors altered plasma cholesterol profile of male offspring, increasing total (13%; *p* < 0.05) and LDL levels (23%; *p* < 0.01) and trending to increase HDL (8%; *p* < 0.1) cholesterol levels. The female offspring glucose levels also increased (8%; *p* < 0.05) by the maternal administration of methyl donor substrates, while total cholesterol also showed a trend to increase in these groups (10%; *p* < 0.1). In this way, previous studies with adult male Wistar rats have described increased plasma cholesterol-related parameters using the same methyl cocktail [17]. However, it is unclear if the increase of both cholesterol fractions had a protective effect and new studies are necessary to unravel the underlying mechanisms. Further analysis of our model could be focused on pancreas development, insulin secretion or glucose tolerance in order to decipher the causes of the increased glucose levels found in female offspring.

**Figure 3.**
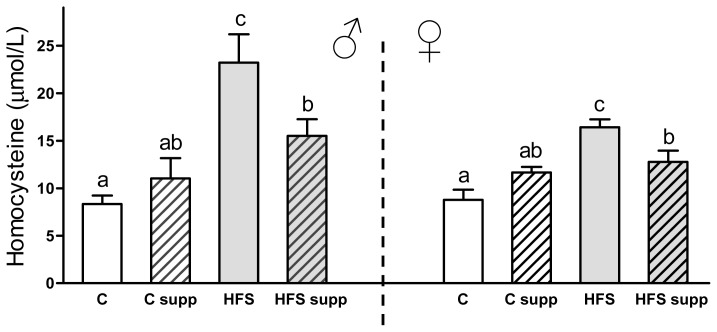
Homocysteine plasma concentration from male and female offspring depending on maternal nutrition during lactation. Data are reported as means ± SE (*n* between 6 to 8 from each experimental group). C, control; C supp, control supplemented; HFS, high-fat-sucrose; HFS supp, high-fat-sucrose supplemented; different letters indicate differences between groups of at least 0.05.

Abnormally high homocysteine plasma levels are strongly associated with increased cardiovascular risk features [27]. In this sense, Figure 3 shows an increase in homocysteine plasma levels due to maternal HFS diet in both sexes (179% in males and 87% in females; *p* < 0.001). Interestingly, maternal methyl donor supplementation reduced this adverse dietary effect and protected against this increase of homocysteine plasma concentrations in both males (−33%; *p* < 0.05) and females (−22%; *p* < 0.05). Stages of high homocysteine rates are usually accompanied by micronutrient deficiencies of vitamin B_12_ and folic acid, as well as with an overweight or obese status [28]. When cardiovascular problems are established, vitamin supplementation can decrease plasma homocysteine concentrations although it is not clear if cardiovascular risk is decreased at the same time [12,29]. For this reason, this novel approach is based on a preventive effect during early life instead of treating an already established stage of hyperhomocysteinemia. Further studies should be focused on the pathology and functionality of the cardiovascular system during adulthood in order to deepen our understanding of the potential role of maternal methyl donor supplementation on the cardiovascular system, and the use of homocysteine as a predisposing factor of disease risk [30,31]. Concerning the possible implicated mechanism, it is unclear if methyl donors exert a direct effect on homocysteine-related metabolic pathways or also regulate homocysteine metabolism by affecting DNA methylation [32,33].

### 2.3. DNA Methylation Metabolism

#### 2.3.1. Hepatic Global DNA Methylation

**Figure 4.**
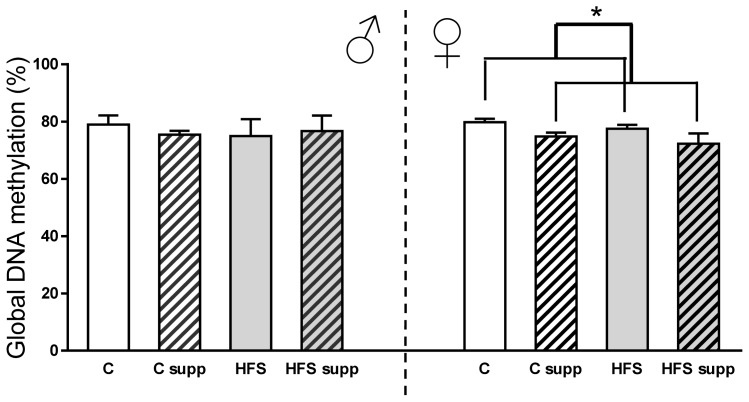
Hepatic global DNA methylation from male and female offspring depending on maternal nutrition during lactation. Data are reported as means ± SE (*n* = 6 from each experimental group). C, control; C supp, control supplemented; HFS, high-fat-sucrose; HFS supp, high-fat-sucrose supplemented; *p < 0.05.

With regard to epigenetic-related features, maternal HFS feeding did not affect hepatic global DNA methylation (Figure 4). However, in the female offspring, maternal methyl donors supplementation decreased liver global DNA methylation (−6%; *p* < 0.05). Decreases in global hepatic DNA methylation due to folic acid supplementation have been previously reported in animal studies [17,34]. However, other studies did not find changes in liver global DNA methylation levels when mothers were supplemented with folic acid [35] or even after six generations of choline, betaine, metionine, zinc, folic acid and vitamin B_12_ supplementation [36]. The effect of these substrates plus an obesogenic diet during perinatal periods influenced the offspring organs and cells in a sex-dependent manner [37]. The absence of changes in global DNA methylation does not necessary mean no changes in DNA methylation profile, since epigenome plasticity could activate methylation and demethylation processes at the same time [38,39]. Although molecular mechanisms underlying global DNA methylation are still uncertain, it is remarkable that promethylating substrates supplemented with the diet are not the final donors of methyl groups, but *S*-adenosylmethionine [40].

#### 2.3.2. Hepatic DNA Methyltransferases mRNA Expression

**Figure 5.**
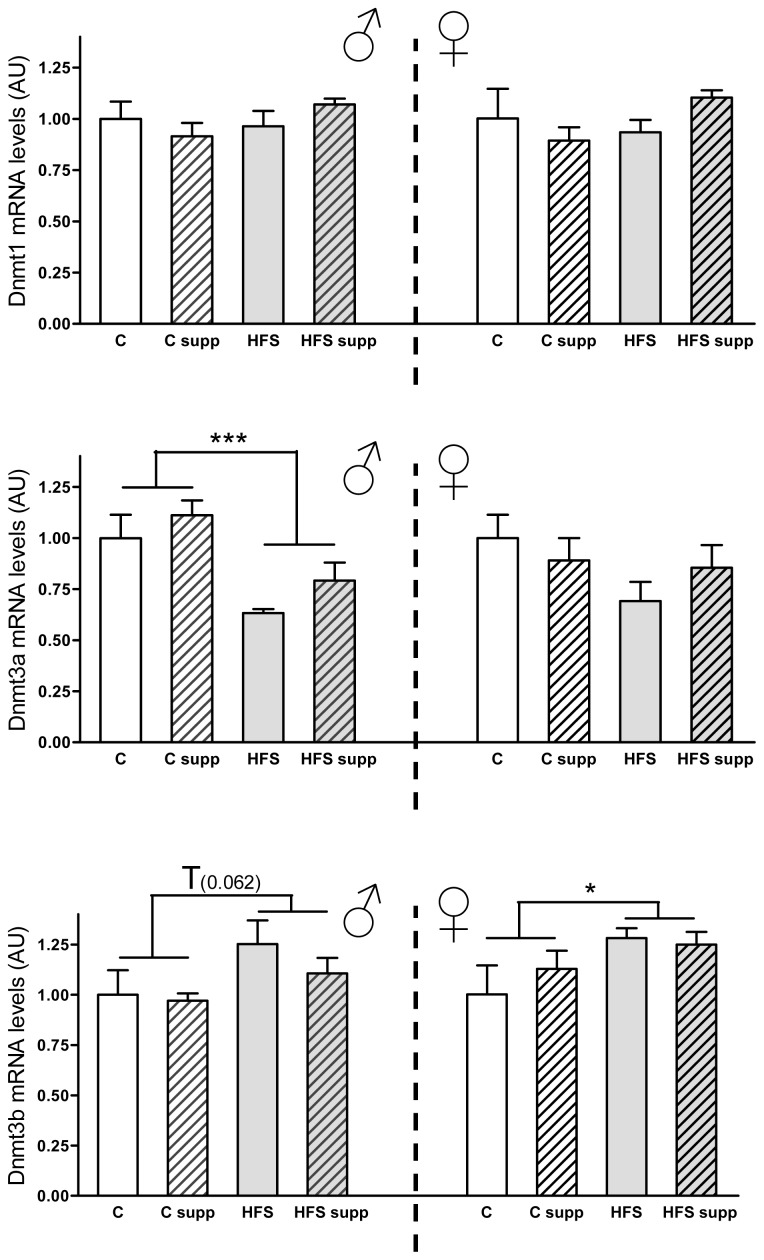
Liver mRNA levels from DNA methyltransferases from male and female offspring depending on maternal nutrition during lactation. Data are reported as means ± SE (*n* = 6 from each experimental group). C, control; C supp, control supplemented; HFS, high-fat-sucrose; HFS supp, high-fat-sucrose supplemented; T, *p* < 0.1; *p < 0.05; ***p < 0.001; AU, Arbitrary Units.

With respect to hepatic mRNA expression of DNA methylation-related genes in the offspring (Figure 5), maternal HFS diet during lactation decreased male *Dnmt3a* (−32%; *p* < 0.001) expression levels and trended to increase those of *Dnmt3b* (20%; *p* = 0.662), which were significantly increased in females (19%; *p* < 0.05). Although *Dnmt3a* and *Dnmt3b* reported opposite expression patterns as a result of maternal obesogenic dietary intake, previous studies have shown distinct final phenotypes and developmental differences in *Dnmt3a* and *Dnmt3b* knock-out animals, suggesting different and independent roles of both enzymes [41,42]. Indeed, other studies have described specific regions preferentially methylated by *Dnmt3a* by using methylation-sensitive restriction fingerprinting [43]. However, mutations in human *Dnmt3b* gene have been linked with immunodeficiency, centromere instability, and facial anomalies (ICF) syndrome [44]. The increase of *Dnmt3a* in both sexes could be related with an activation of one carbon metabolic pathway, which could be associated with hyperhomocysteinemia and increased *S*-adenosylmethionine, which activates DNA methyl transfer-related enzymes [12,19,40].

**Figure 6.**
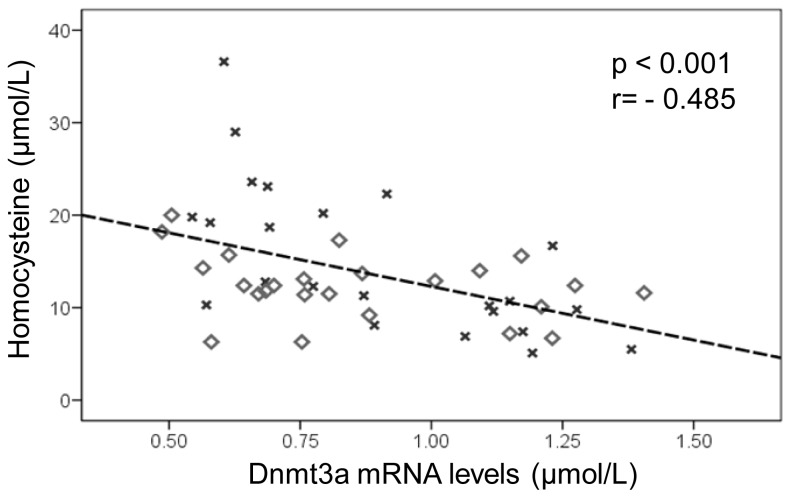
Correlation analysis between plasma homocysteine levels (μmol/L) and hepatic *Dnmt3a* mRNA levels (AU). Male rats are represented by squares and females by crosses.

Finally, It has been previously suggested that liver DNA methylation capacity could be decreased when Aldo-homocysteine intracellular concentration is increased, since this metabolite is associated with plasma hyperhomocysteinemia [12,45]. In this sense, the results of *Dnmt3a* mRNA levels and plasma homocysteine values showed a noteworthy inverse correlation in both sexes (Figure 6) (*r* value = −0.485; *p* < 0.001), reinforcing the association hypothesis between DNA methylation and homocysteine metabolism. Contrariwise, hepatic *Dnmt3b* mRNA levels were not correlated with plasma homocysteine concentrations, which could be due to the different specific roles of both enzymes. Furthermore, the *Dnmt3b* statistical *p* value (marginally significant) and fold changes make it necessary to be cautious with the interpretation of these data.

Although most of the confounding factors influencing the applied animal perinatal models were controlled, there could be additional features that affected the study such as the lack of maternal plasma lipid and homocysteine values or heart histological studies. Other limitations could be the use of the Friedewald equation in animals without an overnight fasting or the difficulty to extrapolate this research model to humans.

## 3. Experimental Section

### 3.1. Animal, Diets and Experimental Design

A total of 32 female twelve-week-old Wistar rats (initial body weight 255 ± 5 g) supplied by the Applied Pharmacobiology Center (CIFA, Pamplona, Spain) were housed at 22 °C with controlled lighting (12 h light-dark cycle) and *ad libitum* access to food and water. They were mated with age-matched Wistar males (CIFA, Pamplona, Spain). After 21 days of pregnancy, excess pups in each litter were removed to keep 8 pups per dam (four males and four females, when possible) as described elsewhere [46] and mothers were randomized to four different dietary groups; control diet (C, *n* = 8), control diet supplemented with methyl donors (C supp, *n* = 8), high-fat-sucrose diet (HFS, *n* = 8) and high-fat-sucrose diet supplemented with methyl donors (HFS supp, *n* = 8). Control diet groups were fed a standard chow diet (2014 Teckland Global 14% Protein Rodent Maintenance Diet, Harlan Iberica, Barcelona, Spain) containing 20% of energy as protein, 67% as carbohydrate (7% as simple sugars) and 13% as lipid, whereas HFS groups were fed an obesogenic diet (D12451, Research Diets, New Brunswick, NJ, USA) containing 20% of energy as protein, 35% as carbohydrates (17% as sucrose) and 45% as lipid. Both diets are designed for a balanced micronutrient composition. Methyl donor supplementation cocktail contains betaine (5 g/kg diet, Sigma Aldrich, St. Louis, MO, USA), choline (5.37 g/kg diet, Sigma Aldrich, St. Louis, MO, USA), folic acid (5.5 mg/kg diet, Sigma Aldrich, St. Louis, MO, USA) and vitamin B_12_ (0.5 mg/kg diet, Sigma Aldrich, St. Louis, MO, USA), as described previously [16–18]. Mothers were fed *ad libitum* during 21 days of lactation. At this time, offspring body weight and length were assessed after anesthesia, whereas total fat mass was measured. At the end of the experimental process (21 days) the offspring were euthanized by decapitation (*n* between 6 to 8 males and females randomly selected 6–7 litters per maternal dietary group), blood collected and plasma stored at −20 °C, and liver was dissected, weighted and stored at −80 °C for later analysis. All the procedures were performed in agreement with the National and Institutional guidelines of the Animal Care and Use Committee at the University of Navarra.

### 3.2. Total Body and Liver Fat Content

Total fat mass was measured by using an EchoMRI analyzer (Echo Medical Systems, Houston, TX, USA), a quantitative magnetic resonance technique [47]. Scans were performed by placing animals into a thin-walled cylinder with a plastic insert added to limit movement. Liver fat content was measured with a special adaptor for tissue sample scanning, as described by the manufacturer (Echo Medical Systems, Houston, TX, USA).

### 3.3. Plasma Analysis

Plasma glucose (HK-CP kit; ABX diagnostics, Montpellier, France), total cholesterol (Cholesterol-CP; ABX diagnostics, Montpellier, France), HDL cholesterol (HDL direct-CP; ABX diagnostics, Montpellier, France), triglycerides (Triglycerides; Randox Laboratories Ltd, Crumlin, UK), free fatty acids (FFA) (NEFA-HR-2 kit; WAKO Chemicals GmbH, Neuss, Germany) and homocysteine levels (Homocysteine Enzymatic Assays; Demeditec Diagnosis, Kiel, Germany) were measured using an automated ABX Pentra C200 equipment (Horiba ABX Diagnostics, Montpellier, France). LDL cholesterol levels were estimated by the Friedewald equation (LDLc = Total cholesterol − HDLc − TG/5) [48].

### 3.4. DNA and RNA Isolation

Liver DNA and RNA were extracted (*n* = 6 animals from 5–6 litters per maternal dietary group) using the QIAamp DNA and RNA mini Kit (Qiagen GmbH, Hilden, Germany). Hepatic DNA concentration was quantified with PicoGreen DNA Quantification Reagent (Invitrogen, Milpites, CA, USA) and RNA levels with a Nanodrop 1000 Spectrophotometer (Thermo & Scientific, Wilmington, DE, USA). RNA was treated with DNase using RNeasy Micro Kit (Qiagen GmbH, Hilden, Germany) and retrotranscripted into cDNA for later analysis with Ambion WT Expression Kit (Ambion, Carlsbad, CA, USA).

### 3.5. Global DNA Methylation

Global DNA methylation was measured using the [3H]dCTP extension assay. Briefly, about 300 ng of genomic DNA (*n* = 6 animals from 5–6 L per maternal dietary group) were digested overnight by triplicate with HpaII or MspI endonuclease enzymes (New England Biolabs, Ipswich, MD, USA). Another aliquot was incubated without enzymes as control. The single nucleotide extension reaction was performed in a 28 μL reaction mix incubated at 56 °C during 1 h containing 280 ng of DNA, 1X PCR Gold Buffer, 1.0 mM MgCl_2_, 0.28 U of Ampitaq Gold polymerase (Applied Biosystems, Houston, TX, USA) and 0.38 μL dCTP[5-3H] (American Radiolabeled Chemicals, Saint Louis, MI, USA). Twenty-five microlitres from each reaction were applied on Whatman DE-81 ion-exchange filters (Whatman, Kent, UK) and air-dried for 1.5 h. Then, filters were washed three times with Sodium-Phosphate buffer (0.5 M, pH 7.0) and radioactivity levels were measured by liquid scintillation counting (Wallmac 1409, Pharmacia, Uppsala, Sweden). Background results were subtracted from enzyme-treated samples and global DNA methylation calculated as previously described [49].

### 3.6. Real-Time qPCR

Quantitative real-time PCR (*n* = 6 animals from 5–6 L per maternal dietary group) was performed by triplicate using ABI PRISM 7900 HT Fast real-time PCR system (Applied Biosystems, Austin, TX, USA) and Taqman primers (Applied Biosystems, Austin, TX, USA) for *Dnmt1* (Rn00709664_m1*), *Dnmt3a* (Rn01027162_g1*) and *Dnmt3b* (Rn01536419_m1). Fold change between groups was calculated using the 2^−ΔΔCt^ method. cDNA integrity was checked with *18S* (Hs99999901_s1) probe and gene expression levels were normalized with respect to *beta-actin* (Rn00667869_m1*) as internal control.

### 3.7. Statistical Analysis

Results are expressed as means ± standard error (SE). Maternal features during pregnancy and before lactation were assessed using one way ANOVA. Offspring outcomes were assessed using 2 × 2 factorial ANOVA tests (Diet × Supplementation); effect of obesogenic diet intake (C + C supp *vs.* HFS + HFS supp) plus the effect of Supplementation (C + HFS *vs.* C supp + HFS supp). This statistical test is also able to define interactions between both factors by DMS *post hoc* test for multiple comparisons. Male and female offspring were separately analyzed due to sexual dimorphism. Pearson’s rank correlation was also used to analyze *Dnmt3a* liver mRNA and plasma homocysteine levels potential association. The statistically significant probability was set at *p* < 0.05. All the statistical analyses were performed with SPSS 15.0 program for Windows (SPSS, Chicago, IL, USA).

## 4. Conclusions

In conclusion, maternal high-fat sucrose dietary intake during lactation could program offspring obesity-related physiological features in both sexes. Interestingly, maternal supplementation with a micronutrient cocktail based on methylation donor substrates appears to protect against plasma hyperhomocysteinemia, probably affecting key metabolic enzymes involved in the methionine-homocysteine cycle.

## Figures and Tables

**Table 1.** Maternal perinatal features.

	C	C supp	HFS	HFS supp	ANOVA
Weight before pregnancy (g)	260.8 ± 13.1	269.3 ± 10.2	246.3 ± 10.9	243.8 ± 5.5	n.s.
Weight gain during pregnancy (%)	31.4 ± 1.6	34.0 ± 2.0	34.4 ± 2.4	32.6 ± 2.5	n.s.
Weight after birth (g)	270.5 ± 14.5	282.3 ± 8.7	267.3 ± 11.1	256.9 ± 8.3	n.s.
Intrauterine number of pups	10.1 ± 0.8	11.5 ± 0.6	9.9 ± 0.6	9.8 ± 0.6	n.s.
Intrauterine number of males	5.9 ± 0.8	5.5 ± 0.4	4.6 ± 0.8	4.8 ± 0.6	n.s.
Intrauterine number of females	4.3 ± 0.4	6.0 ± 0.6	5.3 ± 0.4	5.0 ± 0.7	n.s.

Data are reported as means ± SE (*n* = 8 from each experimental group). C, control; C supp, Control supplemented; HFS, high-fat-sucrose; HFS supp, high-fat-sucrose supplemented; n.s., non significative.

**Table 2.** Offspring biochemical plasma values depending on maternal nutrition during lactation.

	C	C supp	HFS	HFS supp	2 × 2 ANOVA		

					DIET	SUPPL	DIET × SUPPL
**Male offspring plasma values**							

Glucose (mg/dL)	141.3 ± 6.1	140.9 ± 1.6	141.2 ± 3.6	151.7 ± 5.9	n.s.	n.s.	n.s.
Total cholesterol (mg/dL)	111 ± 4	122 ± 8	132 ± 7	155 ± 4	***	*	n.s.
HDL cholesterol (mg/dL)	29.0 ± 1.7	31.5 ± 1.9	34.9 ± 1.2	38.1 ± 1.0	***	0.077	n.s.
LDL cholesterol (mg/dL)	65.4 ± 4.9	74.6 ± 6.4	65.1 ± 6.1	88.1 ± 2.0	n.s.	**	n.s.
Triglycerides (mg/dL)	83 ± 12	80 ± 13	109 ± 13	134 ± 14	**	n.s.	n.s.
Free fatty acides (mg/dL)	0.47 ± 0.10	0.45 ± 0.04	0.81 ± 0.11	0.66 ± 0.05	**	n.s.	n.s.

**Females offspring plasma values**							

Glucose (mg/dL)	132.6 ± 4.6	148.8 ± 3.7	141.0 ± 5.4	145.8 ± 3.2	n.s.	*	n.s.
Total cholesterol (mg/dL)	110 ± 3	126. ± 11	142. ± 6	149 ± 4	***	0.063	n.s.
HDL cholesterol (mg/dL)	28.2 ± 1.2	31.9 ± 2.1	34.1 ± 1.6	33.6 ± 0.8	*	n.s.	n.s.
LDL cholesterol (mg/dL)	62.8 ± 2.2	75.6 ± 7.3	80.0 ± 6.1	83.8 ± 7.8	0.051	n.s.	n.s.
Triglycerides (mg/dL)	95 ± 15	91 ± 23	137 ± 30	157 ± 43	0.092	n.s.	n.s.
Free fatty acides (mg/dL)	0.56 ± 0.05	0.36 ± 0.08	0.75 ± 0.09	0.73 ± 0.08	**	n.s.	n.s.

Data are reported as means ± SE (*n* between 6 to 8 from each experimental group). C, control; C supp, Control supplemented; HFS, high-fat-sucrose; HFS supp, high-fat-sucrose supplemented; n.s., non significative;

* *p* < 0.05;

** *p* < 0.01;

*** *p* < 0.001.
